# Effects of 10 Hz Repetitive Transcranial Magnetic Stimulation of the Left Dorsolateral Prefrontal Cortex in Disorders of Consciousness

**DOI:** 10.3389/fneur.2017.00182

**Published:** 2017-05-03

**Authors:** Xiaoyu Xia, Yang Bai, Yangzhong Zhou, Yi Yang, Ruxiang Xu, Xiaorong Gao, Xiaoli Li, Jianghong He

**Affiliations:** ^1^School of Medicine, Tsinghua University, Beijing, China; ^2^Department of Neurosurgery, PLA Army General Hospital, Beijing, China; ^3^Institute of Electrical Engineering, Yanshan University, Qinhuangdao, China; ^4^State Key Laboratory of Cognitive Neuroscience and Learning, IDG/McGovern Institute for Brain Research, Beijing Normal University, Beijing, China

**Keywords:** vegetative state, unresponsive wakefulness syndrome, minimally conscious state, disorders of consciousness, repetitive transcranial magnetic stimulation

## Abstract

**Background:**

While repetitive transcranial magnetic stimulation (rTMS) has been applied in treatment of patients with disorders of consciousness (DOC), a standardized stimulation protocol has not been proposed, and its therapeutic effects are inconsistently documented.

**Objectives:**

To assess the efficacy of rTMS in improving consciousness in patients with persistent minimally conscious state (MCS) or unresponsive wakefulness syndrome (UWS), previously known as vegetative state (VS).

**Method:**

A prospective single-blinded study, with selected subjects, was carried out. In total, 16 patients (5 MCS and 11 VS/UWS) with chronic DOC were included. All patients received active 10 Hz rTMS at the left dorsolateral prefrontal cortex (DLPFC), at one session per day, for 20 consecutive days. A single daily session of stimulation consisted of 1,000 pulses (10 s of 10 Hz trains; repeated 10 times with an inter-train interval of 60 s; and 11 min and 40 s for total session). The main outcome measures were changes in the total score on the JFK Coma Recovery Scale-Revised (CRS-R) scale. Additional measures were the impressions of caregivers after the conclusion of the interventions, which were assessed using the Clinical Global Impression-Improvement (CGI-I) scale.

**Results:**

The CRS-R scores were increased in all 5 MCS patients and 4 of 11 VS/UWS patients, while a significant enhancement of CRS-R scores was observed compared to the baseline in all participants (*p* = 0.007). However, the improvement was more notable in MCS patients (*p* = 0.042) than their VS/UWS counterparts (*p* = 0.066). Based on the CGI-I scores, two patients improved considerably, two improved, six minimally improved, six experienced no change, and none deteriorated. Good concordance was seen between the CGI-I result and the increases in CRS-R scores.

**Conclusion:**

Treatment of 10 Hz multisession rTMS applied to the left DLPFC is promising for the rehabilitation of DOC patients, especially those in MCS. Further validation with a cohort of a larger sample size is required.

## Introduction

Severe chronic disorders of consciousness (DOC) after acute coma, which typically resolves within 2 weeks, mainly consist of two subgroups: unresponsive wakefulness syndrome (UWS), previously known as vegetative state (VS), and minimally conscious state (MCS) (1). VS/UWS is characterized by wakefulness without awareness (2), whereas MCS is characterized by minimal but definite behavioral evidence of self- or environmental awareness (3). Recently, MCS has been subcategorized into minimally conscious state minus (MCS−) and minimally conscious state plus (MCS+), based on the level of non-reflexive responsiveness of the patients concerned (4). MCS− is characterized by non-communicative responses to meaningful stimuli, whereas MCS+ is characterized by command following. This definition is based on the various levels of consciousness retained in different entities and implicates that specific treatment is needed and various prognoses can be expected (5, 6). However, there are still no evidence-based guidelines regarding the treatment of DOC (7), while neurostimulation techniques have been seen as potential experimental approaches to DOC treatment (8–10). As these invasive methods, including deep brain stimulation and spinal cord stimulation, have ethical and procedural limitations (11), extensive developments have recently been made in approaches that deploy non-invasive brain stimulation (NIBS).

One of these is transcranial magnetic stimulation (TMS), which is a safe, non-invasive, and painless technique that can be applied one stimulus at a time (single-pulse TMS), in pairs of stimuli separated by a variable interval (paired-pulse TMS) or in trains repetitive transcranial magnetic stimulation (rTMS) (12). When TMS is given repetitively, it has been shown to have a neuro-modulatory effect. Repetitive TMS involves delivery of repeated single-pulse stimulation to specific brain regions (13) and has been shown to alter cortical excitability, an effect that outlasts the period of the stimulation (14). Despite intra- and interindividual variability of responses to rTMS (15), low-frequency rTMS (~1 Hz) has been shown to reduce cortical excitability, while the high-frequency variety (5–20 Hz) has been demonstrated to increase it (16). Attempts to use rTMS for DOC treatment have been reported, although the clinical efficacy of this new therapy remains ill defined (17–21).

The induced effects of rTMS are related to the site of stimulation, which varied in previous studies. The primary motor cortex (M1) (18–20) and the dorsolateral prefrontal cortex (DLPFC) (17, 21) are the most commonly chosen stimulation sites. The rationale has been to improve either motor or cognitive function (22). The DLPFC is thought to play a central and integrative function for motor control and behavior and is also a critical component of the decision-making network (23). Meanwhile, magnetic stimulation of the DLPFC has been found to improve learning and memory (24). Several researchers have chosen the right DLPFC, rather than the left, as the site of stimulation (17, 21). The right DLPFC has been linked to maintenance of sustained arousal and attention, as it has strong connections with the reticular formation (25), which is similarly relevant for patients with DOC. However, one previous study showed that a single session of 10 Hz rTMS over the right DLPFC did not induce, at group level, any clinical improvement or intra-/intercortical connectivity changes (21). The left DLPFC area receives visual and somatosensory input from the parietal heteromodal association cortices regarding vision, motion, spatial orientation, and tactile sensations and projects to subcortical, monoaminergic, and cholinergic sources (26). Previous studies have shown that high-frequency rTMS over the left DLPFC has beneficial effects on the linguistic and cognitive skills of patients with Alzheimer’s disease (27). There is also evidence that rTMS of the left DLPFC could improve major depressive disorder as caused by Parkinson’s disease (28). This approach was also selected in a range of transcranial direct current stimulation studies on DOC patients (22, 26). Based on all the above considerations, we opted to stimulate the left DLPFC in this study.

In this research, we proposed a new stimulation protocol consisting of 10 Hz L-DLPFC rTMS and applied it in patients with VS/UWS or MCS. We aimed to verify whether repeated sessions of 10 Hz rTMS, delivered to the left DLPFC, may produce clinically useful behavioral modifications in DOC patients.

## Materials and Methods

### Study Participants

All participants came from the Department of Neurosurgery, PLA Army General Hospital and were recruited from December 2015 to June 2016. The hospital is one of the major treatment centers for DOC in Beijing. All patients enrolled in this study had been of DOC status for more than 3 months according to the JFK Coma Recovery Scale-Revised (CRS-R) scores, which are widely used to define the level of consciousness and to monitor neurobehavioral recovery in patients. The CRS-R scale is based on six subscales that address the auditory, visual, motor, oromotor/verbal, communication, and arousal processes (29). All patients recruited in this study received a routine medication and rehabilitation course after admission to hospital. They were monitored closely, and those who were stabilized with no consciousness improvement for 1 month received rTMS protocol in the following month. Patients who showed an obvious increase or decrease in consciousness before rTMS treatment were excluded, as were those who experienced complications such as acute pneumonia during rTMS treatment. Participants with a history of epilepsy, or who had or had had pacemakers, intrathecal baclofen pumps, hydrocephalus shunts, aneurysm clips, electrodes, or other devices implanted were also eliminated. Routine medicine was delivered orally through a nasogastric tube.

### Study Design and Stimulation Protocol

**Figure 1:**
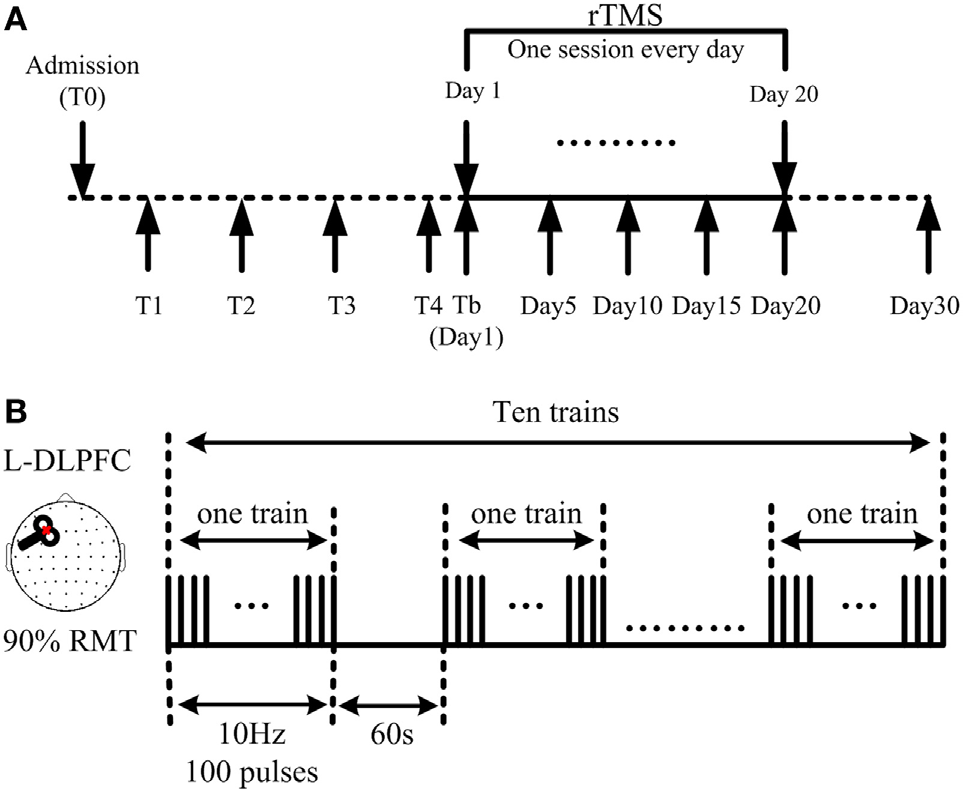
**Demonstration of the repetitive transcranial magnetic stimulation (rTMS) protocol**. **(A)** 10 Hz rTMS was delivered at the left dorsolateral prefrontal cortex (DLPFC), lasting for 20 consecutive days. Coma Recovery Scale-Revised scores were assessed at days 1, 5, 10, 15, 20, and 30. **(B)** Illustration of one session: a single daily session of stimulation consisted of 1,000 pulses; 10 s as one train; 10 trains as one session, interrupted with an interval of 60 s; 11 min and 40 s for one session.

After a routine medication and rehabilitation course in the first month of their admission to hospital, all stabilized patients who had no consciousness improvement during said month received active 10 Hz rTMS at the left DLPFC for one session per day over 20 consecutive days (Figure 1A). TMS pulses were delivered using a Magstim *R*2 stimulator and an eight-shaped coil (Magstim Company Limited, Whitland, UK), which can produce a biphasic waveform in a pulse width of ~0.1 ms. The coil was placed tangentially toward the scalp over the left DLPFC (position F3 of the 10/20 international electroencephalography system) for active stimulation. Stimulation intensity was determined based on the resting motor threshold (RMT) for each patient, which was defined as the lowest TMS intensity to evoke at least 5 out of 10 EMG with amplitude larger than 50 µV peak-to-peak in the relaxed first dorsal interosseous muscle of the right hand, according to the IFCN Committee recommendations (30). A single daily session of stimulation consisted of 1,000 pulses (10 Hz trains for 10 s; repeated 10 times with an inter-train interval of 60 s; 11 min and 40 s for total session) at an intensity of 90% RMT (Figure 1B). Inserted earplugs, which continuously played a masking noise, were used to prevent the induction of auditory potentials by the click associated with TMS discharge. Bone conduction was attenuated by placing a thin layer of foam between the coil and scalp. Meanwhile, patients’ routine medication and rehabilitation courses continued as usual during rTMS treatment.

### Outcome Measures

Clinical evaluations using the CRS-R were performed at five different time points during and after rTMS treatment, including at days 5, 10, 15, and 20, with the final assessment being on day 30, 10 days after completion of the rTMS protocol (Figure 1A). All CRS-R assessments were undertaken by a single trained clinician who was blinded to this study.

The Clinical Global Impression-Improvement (CGI-I) scale (31) was applied on day 30 by family members who had been assisting their relatives at the bedside for at least 3 months. It is commonly used as an outcome measure in studies that are evaluating the efficacy of medical treatments (CGI-I score interpretation: 1 = much improved, 2 = improved, 3 = minimally improved, 4 = no change, 5 = minimally worse, 6 = worse, 7 = much worse).

Side effects were also monitored during the study, including seizure induction and scalp burns.

### Statistics

The Mann–Whitney *U* test was used to compare the baseline consciousness between the two groups of patients, while the Wilcoxon signed-rank test was deployed to analyze the therapeutic effects of our treatment protocol. *p* < 0.05 was recognized as significant. Spearman’s rank correlation coefficient was calculated to assess the correlation between the CRS-R scale and the CGI-I measurements.

## Results

This study featured a total of 41 DOC patients, who were hospitalized in the ward where we conducted our investigations from December 2015 to June 2016. During their routine medication and rehabilitation course in the first month of their admission, 16 patients remained in a stable clinical state and displayed no consciousness improvement. Eventually, all of these (ranging from 23 to 67 years old, including five women,) completed the study. There was no focal lesion in the left DLPFC in any of said participants, as evidenced by the brain scans. Detailed demographic and clinical characteristics of these patients are reported in Table 1. Based on the CRS-R scores, 5 patients were classified as MCS and the other 11 as VS/UWS.

**Table 1:** **Demographic and clinical characteristics of all the DOC patients included in the study**.

Patient	Sex	Age	Etiology	Duration (months)	CRS-R							Diagnosis	Treatment
					A	V	M	OM	C	Ar	Total		
P1	M	23	TBI	13	1	0	3	1	0	2	7	MCS−	Amantadine, Baclofen
P2	F	47	Stroke (ICH)	6	1	1	3	1	0	2	8	MCS−	Amantadine
P3	F	31	Anoxia	35	2	2	2	1	0	2	9	MCS−	Amantadine
P4	M	44	Stroke (ICH)	3	1	3	2	1	0	2	9	MCS−	Amantadine, Baclofen
P5	M	47	Stroke (ICH)	3	0	2	2	1	0	2	7	MCS−	Amantadine
P6	M	67	Stroke (ICH)	4	1	1	2	1	0	0	5	VS	Amantadine, Baclofen
P7	F	26	Stroke (ICH)	4	1	0	2	1	0	2	6	VS	Amantadine, Baclofen
P8	M	39	Stroke (ICH)	4	1	1	2	1	0	2	7	VS	Amantadine, Baclofen
P9	M	40	TBI	16	1	0	2	1	0	2	6	VS	Amantadine, Baclofen
P10	M	27	Stroke (ICH)	11	0	0	2	1	0	1	4	VS	Amantadine
P11	M	52	Stroke (ICH)	4	1	1	2	1	0	0	5	VS	Amantadine
P12	M	60	Anoxia	3	1	0	2	1	0	2	6	VS	Amantadine, Baclofen
P13	M	42	Stroke (CI)	6	1	1	2	1	0	2	7	VS	Amantadine
P14	M	35	Anoxia	3	0	0	2	1	0	2	5	VS	Amantadine
P15	F	51	Anoxia	6	1	0	2	1	0	2	6	VS	Amantadine, Baclofen
P16	F	50	Anoxia	8	1	0	2	1	0	2	6	VS	Amantadine, Baclofen

*Duration: time at which the patient has been in a disorder of consciousness; diagnosis: CRS-R categorization at the time of recruitment*.

*Gray shades were used to distinguish between VS and MCS group patients*.

*CRS-R, Coma Recovery Scale-Revised; VS, vegetative state; ICH, intracerebral hemorrhage; CI, cerebral infarction; MCS, minimally conscious state; F, female; M, male; A, auditory; V, visual; M, motor; OM, oromotor; C, communication; Ar, arousal; DOC, disorders of consciousness*.

### Effects of the 10 Hz rTMS Treatment Protocol As Measured by CRS-R at Day 30

**Figure 2:**
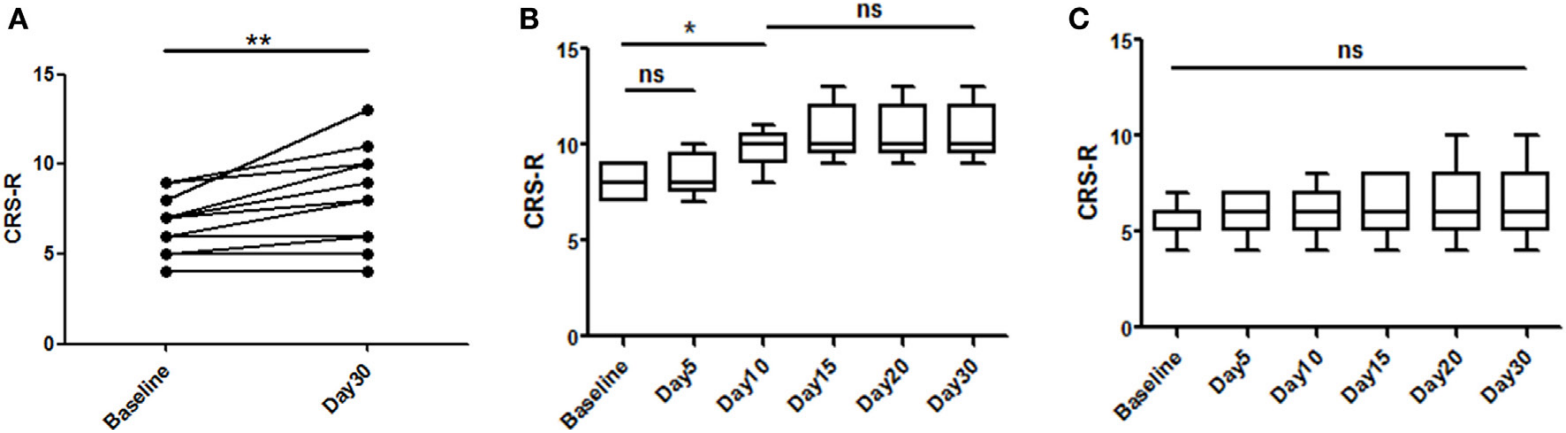
**Effects of repetitive transcranial magnetic stimulation treatment evaluated with Coma Recovery Scale-Revised (CRS-R) in both minimally conscious state (MCS) and VS/UWS patients**. Significant improvement is shown in the entire group [**(A)**, *p* = 0.007]. However, subgroup analysis of the therapeutic effects at each time point only revealed significant differences in the MCS patients [**(B)**, *p* = 0.042], and significant improvement was first shown at day 10 [**(B)**, *p* = 0.041]. In the VS/UWS patients, there was no significant improvement in CRS-R scores [**(C)**, *p* = 0.066]. Data are displayed as the box–whiskers graphs, with the whiskers representing the minimum and maximum of the datasets.

A total of 16 patients with chronic DOC (5 MCS and 11 VS/UWS) completed the treatment (Table 1; Figure 1), with no specific side effects observed. At day 30, a significant increase in the CRS-R scores was observed compared to the baseline in all participants (Figure 2A, *p* = 0.007). Specifically, diagnoses were changed in five patients, based on their CRS-R scores (Table 2). We also analyzed said scores separately based on the baseline diagnosis and found significant improvement in the MCS patients (*p* = 0.042) rather than in their VS/UWS counterparts (*p* = 0.066) (Figures 2B,C).

**Table 2:** **Clinical evaluation of the DOC patients at day 30**.

Patient	CRS-R improvement							Change of diagnosis
	A	V	M	OM	C	Ar	Total	
P1	0	2	0	0	0	0	2(+)	Remained MCS−
P2	3	2	0	0	0	0	5(+)	MCS− elevated to MCS+
P3	0	1	1	0	0	0	2(+)	Remained MCS−
P4	1	0	0	0	0	0	1(+)	Remained MCS−
P5	3	0	0	0	0	0	3(+)	MCS− elevated to MCS+
P6	0	0	0	0	0	0	0	Remained VS/UWS
P7	0	0	0	0	0	0	0	Remained VS/UWS
P8	2	0	1	0	0	0	3(+)	VS/UWS elevated to MCS+
P9	0	0	0	0	0	0	0	Remained VS/UWS
P10	0	0	0	0	0	0	0	Remained VS/UWS
P11	0	0	0	0	0	0	0	Remained VS/UWS
P12	0	0	0	0	0	0	0	Remained VS/UWS
P13	0	0	1	0	0	0	1(+)	VS/UWS elevated to MCS−
P14	1	0	0	0	0	0	1(+)	Remained VS/UWS
P15	2	0	0	0	0	0	2(+)	VS/UWS elevated to MCS+
P16	0	0	0	0	0	0	0	Remained VS/UWS

*Gray shades were used to distinguish between VS and MCS group patients*.

*A, auditory; V, visual; M, motor; OM, oromotor; C, communication; Ar, arousal; DOC, disorders of consciousness; CRS-R, Coma Recovery Scale-Revised; MCS, minimally conscious state*.

### Specific Clinical Manifestations Related to CRS-R Score Elevation

The CRS-R scores were increased in all five MCS patients. P1 had a baseline CRS-R score of 7; his score increased because he recovered the ability to visually pursue his own reflection in a moving mirror; the patient’s status remained as MCS−. P2 had a baseline CRS-R score of 8. Accompanied by stimulation, the consciousness level of the patient rose from MCS− to MCS+, while the CRS-R score was elevated from 8 to 13. P3 had a baseline CRS-R score of 9, which finally increased to 11. This augmentation was based on the participant’s ability to localize noxious stimulation and visually pursue bright images on a mobile phone screen. Moving on to P4, this patient had a baseline CRS-R score of 9, which increased slightly to 10 as he recovered the ability to localize sound. P5, meanwhile, had a baseline CRS-R score of 7, which was boosted to 10 as a result of the participant regaining the faculty of following commands reproducibly; the subject was then diagnosed as MCS+.

Turning to the other group, the CRS-R scores increased in 4 of 11 VS/UWS patients. First, P8 had a baseline CRS-R score of 7. His score increased to 10 due to improvement in auditory and motor function, including the ability to localize noxious stimulation and follow commands reproducibly; consequently, his status was improved to MCS+. P13 also had a baseline CRS-R score of 7, which was nudged up to 8 as he regained the ability to pinpoint noxious stimulation; the patient’s status was elevated to MCS−. P14, meanwhile, had a baseline CRS-R score of 5, which rose slightly to 6 due to an improvement in his auditory scale; his status remained as VS/UWS. Finally, P15 had a baseline CRS-R score of 6. This increased to 8, as she recovered the ability to follow commands reproducibly; her status was redefined as MCS+.

### Onset Time of the Therapeutic Effect in the MCS Patients

We analyzed the CRS-R scores at all time points and found that day 10 was the earliest point at which we could observe a significant improvement in the MCS patients (*p* = 0.041) (Figure 2B). However, the patients stabilized and no significant improvement was observed after day 10.

### CGI-I Evaluations by Caregivers and Correlation between the CRS-R Scores and CGI-I Scores

**Figure 3:**
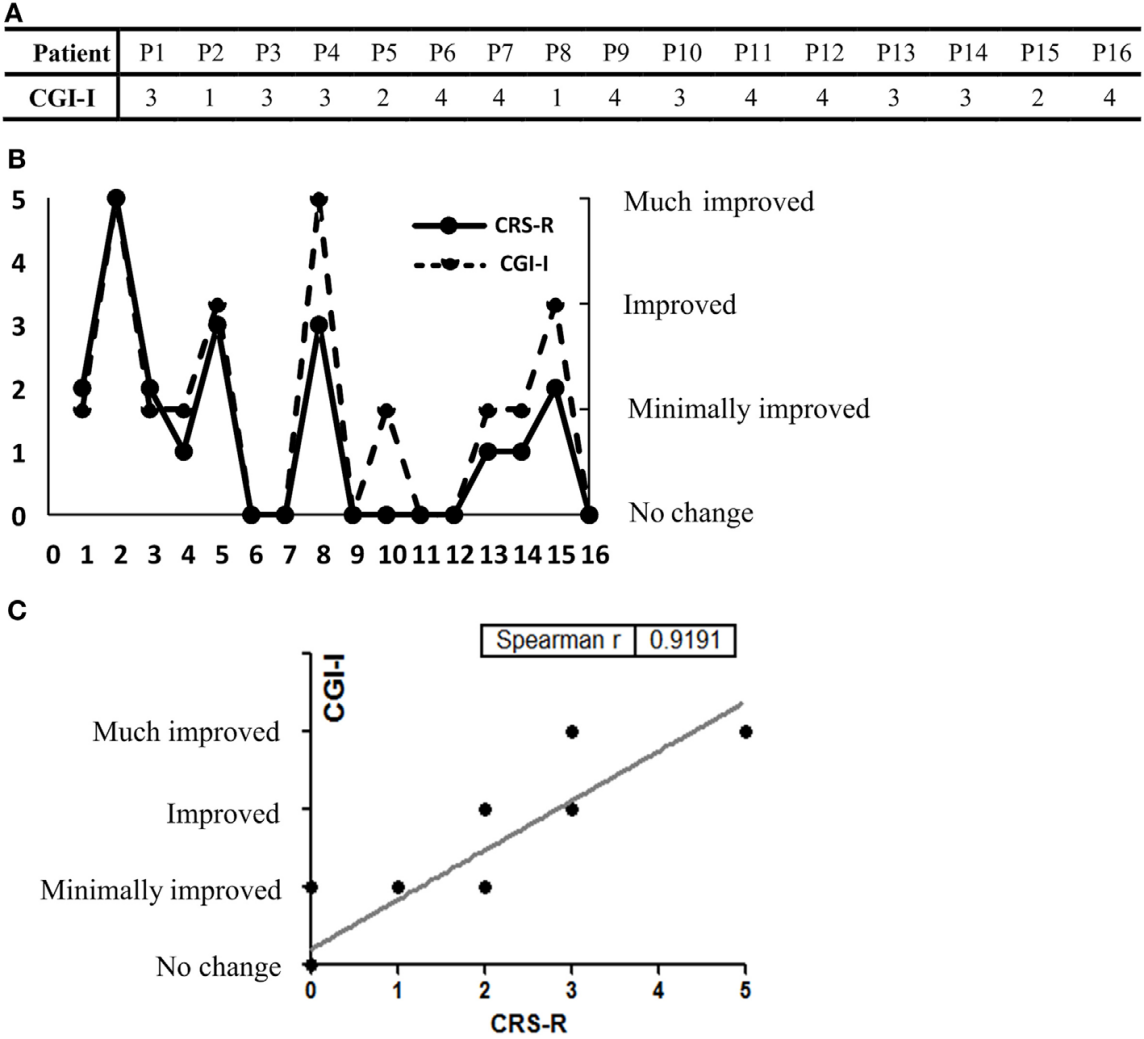
**(A)** The Clinical Global Impression-Improvement (CGI-I) scores of all 16 participants are listed. **(B)** CGI-I evaluations and the Coma Recovery Scale-Revised (CRS-R) score elevation at day 30. **(C)** Good concordance was seen between the results of the two rating scales (Spearman’s *r* = 0.9191).

Clinical Global Impression-Improvement scores were listed (Figure 3A). We found similar results from both scales in all patients in terms of improvement posttreatment (Figure 3B). In addition, correlation analysis showed good correlation between the CRS-R and CGI-I scores (Spearman’s *r* = 0.9191) (Figure 3C).

## Discussion

This clinical study demonstrated that 10 Hz multisession rTMS applied to the left DLPFC could produce beneficial behavioral modifications, as tested by the CRS-R and CGI-I scales in DOC patients, without observable side effects. MCS patients may benefit more from this treatment than VS/UWS patients on a group level.

As a representative of NIBS approaches, TMS has attracted increasing attention in the DOC research field. There is an emerging possibility of using rTMS in an attempt to treat DOC patients, with some studies reporting encouraging results (17, 18, 21). Louise-Bender Pape et al. (17) reported a trend toward significant neurobehavioral gains that were temporally related to patterned rTMS of the right DLPFC in a traumatic VS/UWS patient. Piccione et al. (18), meanwhile, reported an arousal with transient increase of meaningful behaviors in an MCS patient following a single session of 20 Hz rTMS of the M1. Finally, Naro et al. (21) demonstrated that a single session of 10 Hz rTMS over the right DLPFC may transiently improve consciousness and partially restore the connectivity within several cortical areas, based on positive results in 3 of 10 postanoxic VS/UWS patients. However, there have also been reports of negative results (19, 20). An uncontrolled study performed by Manganotti et al. (19) found no clinical modifications in three VS and two MCS patients using the rTMS paradigm of Piccione et al. (18), while long-lasting behavioral and neurophysiological modifications were observed in only one MCS patient with rTMS over M1. Elsewhere, a randomized, sham-controlled study performed by Cincotta et al. (20) showed no effects of 20 Hz rTMS on the primary motor cortex in VS patients. Another confounding factor is that among all the studies reported, stimulation protocols varied widely. There is no specific, unified criterion regarding rTMS for DOC treatment. Therefore, its clinical application still requires deep investigation.

We designed our stimulation protocol on the basis of full consideration and a literature review. In addition to the stimulation site mentioned earlier, stimulation frequency and duration may also be relevant to the final effect. Low-frequency rTMS can induce inhibition in neural activity, while the high-frequency variety can activate neurons (32, 33). Seizure induction after 20 Hz rTMS stimulation was applied to the DLPFC had been reported earlier (34). No adverse events were caused in participants in a single-session 10 Hz DLPFC rTMS study. Given the current level of evidence regarding rTMS for DOC, and taking safety considerations into account, we chose 10 Hz as the effective stimulation frequency. Naro et al. (21) demonstrated that a single session of 10 Hz rTMS brought a significant, but transient, clinical improvement in only 3 of 10 VS/UWS patients in a similar study. Just one session may not be sufficient for a treatment protocol. The protocol used in this study was a revision based on Naro’s study (21); we used 20 sessions, rather than just 1, to verify the accumulation efficacy of the rTMS. A multisession protocol seems more effective than that with only one session. However, it is not certain that the effect will remain at the same level over prolonged treatment. In the current study, significant improvement was first shown at day 10, while no significant improvement was noticed after that date in the MCS set. The duration of the stimulation protocol will require further exploration.

While repetitive TMS has been administered to DOC patients for some time, there is insufficient evidence to formulate recommendations regarding their use in clinical practice (1). An obvious study difficulty is how to disentangle variations related to natural recovery or other treatment from rTMS-induced changes. The strongest design for supporting our hypothesis would be a parallel group design wherein some subjects receive sham treatment and others real treatment. However, the etiologies and clinical statuses are highly heterogeneous among DOC patients. There are logistical and methodological difficulties associated with conducting placebo-controlled trials in this population (35), especially with limited subjects. We used a self-controlled study before and after rTMS treatment. Restrictions were imposed on the selection criteria of participants in an attempt to minimize interference with natural recovery. All measures were taken to improve the credibility of the results. Although the study design was far from ideal, the results still gave us many meaningful pointers.

Our study illustrates the residual capacity for neural plasticity and recovery of consciousness in some DOC patients. If we separate VS/UWS and MCS as two groups, the curative effect observed is significantly different between them. MCS patients can attain more benefit from rTMS on a group level. This is in line with previous studies that showed greater capacity for neural plasticity in patients in MCS (22, 36, 37). However, it is worth noting that a minority of our VS/UWS patients also improved behaviorally, with three even being elevated to MCS. This implies that neural networks are capable of reacting as an efficient substrate for the remote effects of rTMS, which were also retained in at least some VS/UWS participants. The degree of disturbance of consciousness is more like a continuous spectrum, and there may be no absolute discernible boundaries between VS/UWS and MCS. The reasons for the negative results of the current rTMS trial in other VS/UWS patients are complicated; they may be related to a much more severe impairment of functional cortical connectivity in VS/UWS. Another hypothesis is that the rTMS intensity employed may have been too low. Perhaps in some situations, cortical connectivity is completely (or almost completely) destroyed, so that rTMS can cause no reaction. It is also reasonable to speculate that in certain patients, cortical connectivity is retained to some extent. While it is possible that higher rTMS intensity could produce some effect, this has not yet been verified. Further investigations are required.

To the best of our knowledge, although the sample size was still very small, this was the first study for DOC therapy to suggest that rTMS over DLPFC may induce clinical improvement at group level; it also involved more subjects than previous reports. Some of the latter were case reports, with only one patient discussed (17, 18). The numbers of subjects in other previous studies were all very small. For example, Manganotti et al. (19) included three VS and three MCS patients, finding long-lasting behavioral and neurophysiological modifications in only one MCS subject. Naro et al. (21) included 10 postanoxic VS/UWS patients and found a significant, but transient, clinical improvement in only 3. Meanwhile, Cincotta et al. (20) included 11 VS/UWS patients and reported a negative result. Our study included 5 MCS and 11 VS/UWS participants, and we found that 4 and 5 improved respectively. In addition, the effect of the current stimulation protocol appears more definite when compared with previous studies. We found statistically significant results, especially for MCS patients. Although a series of treatments are routinely administered for DOC patients, few interventions have been rigorously shown to be effective (38). To date, the evidence has been insufficient to make rTMS—an established method for DOC patients in clinical practice (1). Although our preliminary results still could not be used to formulate a recommended, specific and unified criterion regarding rTMS for DOC treatment, they add evidence to the case for rTMS as an effective method of neurorehabilitation. As the effect of this multisession protocol appeared to be better than that of only one session, the accumulation efficacy of rTMS has been partly verified. The long-term effect needs to be verified to determine its clinical and practical value in future work. Extended follow-up time and prolonged or repeated stimulation should be considered. A larger study with controlled subjects, which meets rigorous inclusion and exclusion criteria and follows a carefully defined protocol using several methods of evaluating consciousness, both global and specific, would be needed.

## Conclusion

We demonstrated that 10 Hz multisession rTMS applied to the left DLPFC seems promising for the rehabilitation of patients with severe DOC. Beneficial effects were observed in all MCS patients, and some VS/UWS patients, at the individual level. Significant improvement was found in the former, rather than the latter, at group level. Due to the relatively small sample size, patients in this study were not subdivided and discussed according to brain injury etiology. We cannot confidently state that an optimal stimulation protocol has been found and confirmed for DOC treatment. Further studies are needed to verify the clinical effect of rTMS on larger numbers of patients. The implication of brain injury etiology for rTMS treatment could also be studied.

## Ethics Statement

The study was conducted according to the Declaration of Helsinki and approved by the local ethics committee of PLA Army General Hospital. All patients were admitted to the Department of Neurosurgery at this hospital, where all procedures took place. Given their inability, we obtained written informed consent from each participant’s legal surrogate.

## Author Contributions

Full access to all the data in the study and responsibility for the integrity of the data and the accuracy of the data analysis; administrative, technical, or material support: XX. Study concept and design; acquisition, analysis, or interpretation of data: YB and XX. Drafting of the manuscript; statistical analysis: YB. Critical revision of the manuscript for important intellectual content: all authors. Obtained funding: XX, YY, JH and XL. Study supervision: RX, XG, JH, and XL.

## Conflict of Interest Statement

The authors declared no potential conflicts of interest with respect to the research, authorship, and/or publication of this manuscript. And they will not participate in state-sponsored commercialization of the research findings.

## References

[1] Giacino JT Fins JJ Laureys S Schiff ND. Disorders of consciousness after acquired brain injury: the state of the science. Nat Rev Neurol (2014) 10:99–114. 10.1038/nrneurol.2013.279 24468878

[2] Jennett B Plum F Persistent vegetative state after brain damage. A syndrome in search of a name. Lancet (1972) 1:734–7. 10.1016/S0140-6736(72)90242-5 4111204

[3] Giacino JT Ashwal S Childs N Cranford R Jennett B Katz DI The minimally conscious state: definition and diagnostic criteria. Neurology (2002) 58:349–53. 10.1212/WNL.58.3.349 11839831

[4] Bruno MA Majerus S Boly M Vanhaudenhuyse A Schnakers C Gosseries O Functional neuroanatomy underlying the clinical subcategorization of minimally conscious state patients. J Neurol (2012) 259:1087–98. 10.1007/s00415-011-6303-7 22081100

[5] Giacino JT Kalmar KA The vegetative and minimally conscious states: a comparison of clinical features and functional outcome. J Head Trauma Rehabil (1997) 12:36–51. 10.1097/00001199-199708000-00005

[6] Nakase-Richardson R Whyte J Giacino JT Pavawalla S Barnett SD Yablon SA Longitudinal outcome of patients with disordered consciousness in the NIDRR TBI Model Systems Programs. J Neurotrauma (2012) 29:59–65. 10.1089/neu.2011.1829 21663544

[7] Bernat JL D’Alessandro AM Port FK Bleck TP Heard SO Medina J Report of a National Conference on Donation after cardiac death. Am J Transplant (2006) 6:281–91. 10.1111/j.1600-6143.2005.01194.x 16426312

[8] Yamamoto T Katayama Y Oshima H Fukaya C Kawamata T Tsubokawa T Deep brain stimulation therapy for a persistent vegetative state. Acta Neurochir Suppl (2002) 79:79–82. 10.1007/978-3-7091-6105-0_18 11974994

[9] Schiff ND Giacino JT Kalmar K Victor JD Baker K Gerber M Behavioural improvements with thalamic stimulation after severe traumatic brain injury. Nature (2007) 448:600–3. 10.1038/nature06041 17671503

[10] Yamamoto T Katayama Y Obuchi T Kobayashi K Oshima H Fukaya C. Deep brain stimulation and spinal cord stimulation for vegetative state and minimally conscious state. World Neurosurg (2013) 80: S30.e1–9. 10.1016/j.wneu.2012.04.010 22543046

[11] Giacino J Fins JJ Machado A Schiff ND Central thalamic deep brain stimulation to promote recovery from chronic posttraumatic minimally conscious state: challenges and opportunities. Neuromodulation (2012) 15:339–49. 10.1111/j.1525-1403.2012.00458.x 22624587

[12] Guerra A Costantini EM Maatta S Ponzo D Ferreri F. Disorders of consciousness and electrophysiological treatment strategies: a review of the literature and new perspectives. Curr Pharm Des (2014) 20:4248–67. 10.2174/13816128113196660648 24025061

[13] Hallett M Wassermann EM Pascual-Leone A Valls-Sole J Repetitive transcranial magnetic stimulation. The International Federation of Clinical Neurophysiology. Electroencephalogr Clin Neurophysiol Suppl (1999) 52:105–13. 10590981

[14] Maeda F Keenan JP Tormos JM Topka H Pascual-Leone A. Modulation of corticospinal excitability by repetitive transcranial magnetic stimulation. Clin Neurophysiol (2000) 111:800–5. 10.1016/S1388-2457(99)00323-5 10802449

[15] Fitzgerald PB Fountain S Daskalakis ZJ. A comprehensive review of the effects of rTMS on motor cortical excitability and inhibition. Clin Neurophysiol (2006) 117:2584–96. 10.1016/j.clinph.2006.06.712 16890483

[16] Gorsler A Baumer T Weiller C Munchau A Liepert J. Interhemispheric effects of high and low frequency rTMS in healthy humans. Clin Neurophysiol (2003) 114:1800–7. 10.1016/S1388-2457(03)00157-3 14499741

[17] Louise-Bender Pape T Rosenow J Lewis G Ahmed G Walker M Guernon A Repetitive transcranial magnetic stimulation-associated neurobehavioral gains during coma recovery. Brain Stimul (2009) 2:22–35. 10.1016/j.brs.2008.09.004 20633400

[18] Piccione F Cavinato M Manganotti P Formaggio E Storti SF Battistin L Behavioral and neurophysiological effects of repetitive transcranial magnetic stimulation on the minimally conscious state: a case study. Neurorehabil Neural Repair (2011) 25:98–102. 10.1177/1545968310369802 20647501

[19] Manganotti P Formaggio E Storti SF Fiaschi A Battistin L Tonin P Effect of high-frequency repetitive transcranial magnetic stimulation on brain excitability in severely brain-injured patients in minimally conscious or vegetative state. Brain Stimul (2013) 6:913–21. 10.1016/j.brs.2013.06.006 23928101

[20] Cincotta M Giovannelli F Chiaramonti R Bianco G Godone M Battista D No effects of 20 Hz-rTMS of the primary motor cortex in vegetative state: a randomised, sham-controlled study. Cortex (2015) 71:368–76. 10.1016/j.cortex.2015.07.027 26301875

[21] Naro A Russo M Leo A Bramanti P Quartarone A Calabro RS. A single session of repetitive transcranial magnetic stimulation over the dorsolateral prefrontal cortex in patients with unresponsive wakefulness syndrome: preliminary results. Neurorehabil Neural Repair (2015) 29:603–13. 10.1177/1545968314562114 25539781

[22] Angelakis E Liouta E Andreadis N Korfias S Ktonas P Stranjalis G Transcranial direct current stimulation effects in disorders of consciousness. Arch Phys Med Rehabil (2014) 95:283–9. 10.1016/j.apmr.2013.09.002 24035769

[23] Heekeren HR Marrett S Ruff DA Bandettini PA Ungerleider LG. Involvement of human left dorsolateral prefrontal cortex in perceptual decision making is independent of response modality. Proc Natl Acad Sci U S A (2006) 103:10023–8. 10.1073/pnas.0603949103 16785427 PMC1479865

[24] Muri RM Vermersch AI Rivaud S Gaymard B Pierrot-Deseilligny C. Effects of single-pulse transcranial magnetic stimulation over the prefrontal and posterior parietal cortices during memory-guided saccades in humans. J Neurophysiol (1996) 76:2102–6. 8890321 10.1152/jn.1996.76.3.2102

[25] Sturm W Willmes K On the functional neuroanatomy of intrinsic and phasic alertness. Neuroimage (2001) 14:S76–84. 10.1006/nimg.2001.0839 11373136

[26] Thibaut A Bruno MA Ledoux D Demertzi A Laureys S. tDCS in patients with disorders of consciousness: sham-controlled randomized double-blind study. Neurology (2014) 82:1112–8. 10.1212/WNL.0000000000000260 24574549

[27] Bereau M Magnin E Nicolier M Berthet L Dariel E Ferreira S Left prefrontal repetitive transcranial magnetic stimulation in a logopenic variant of primary progressive aphasia: a case report. Eur Neurol (2016) 76:12–8. 10.1159/000447399 27344155

[28] Shin HW Youn YC Chung SJ Sohn YH. Effect of high-frequency repetitive transcranial magnetic stimulation on major depressive disorder in patients with Parkinson’s disease. J Neurol (2016) 263:1442–8. 10.1007/s00415-016-8160-x 27178002

[29] Giacino JT Kalmar K Whyte J. The JFK Coma Recovery Scale-Revised: measurement characteristics and diagnostic utility. Arch Phys Med Rehabil (2004) 85:2020–9. 10.1016/j.apmr.2004.02.033 15605342

[30] Rossini PM Burke D Chen R Cohen LG Daskalakis Z Di Iorio R Non-invasive electrical and magnetic stimulation of the brain, spinal cord, roots and peripheral nerves: basic principles and procedures for routine clinical and research application. An updated report from an I.F.C.N. Committee. Clin Neurophysiol (2015) 126:1071–107. 10.1016/j.clinph.2015.02.001 25797650 PMC6350257

[31] Guy W ECDEU Assessment Manual for Psychopharmacology. Rockville: US DHEW (1976).

[32] Houdayer E Degardin A Cassim F Bocquillon P Derambure P Devanne H. The effects of low- and high-frequency repetitive TMS on the input/output properties of the human corticospinal pathway. Exp Brain Res (2008) 187:207–17. 10.1007/s00221-008-1294-z 18259738

[33] Rossi S Hallett M Rossini PM Pascual-Leone A Safety, ethical considerations, and application guidelines for the use of transcranial magnetic stimulation in clinical practice and research. Clin Neurophysiol (2009) 120:2008–39. 10.1016/j.clinph.2009.08.016 19833552 PMC3260536

[34] Cavinato M Iaia V Piccione F Repeated sessions of sub-threshold 20-Hz rTMS. Potential cumulative effects in a brain-injured patient. Clin Neurophysiol (2012) 123:1893–5. 10.1016/j.clinph.2012.02.066 22405994

[35] Whyte J. Treatments to enhance recovery from the vegetative and minimally conscious states: ethical issues surrounding efficacy studies. Am J Phys Med Rehabil (2007) 86:86–92. 10.1097/PHM.0b013e31802f0434 17251691

[36] Rosanova M Gosseries O Casarotto S Boly M Casali AG Bruno MA Recovery of cortical effective connectivity and recovery of consciousness in vegetative patients. Brain (2012) 135:1308–20. 10.1093/brain/awr340 22226806 PMC3326248

[37] Ragazzoni A Pirulli C Veniero D Feurra M Cincotta M Giovannelli F Vegetative versus minimally conscious states: a study using TMS-EEG, sensory and event-related potentials. PLoS One (2013) 8:e57069. 10.1371/journal.pone.0057069 23460826 PMC3584112

[38] Faugeras F Rohaut B Weiss N Bekinschtein TA Galanaud D Puybasset L Probing consciousness with event-related potentials in the vegetative state. Neurology (2011) 77:264–8. 10.1212/WNL.0b013e3182217ee8 21593438 PMC3136052

